# Learning Data Correction for Myoelectric Hand Based on “Survival of the Fittest”

**DOI:** 10.34133/2021/9875814

**Published:** 2021-12-13

**Authors:** Yusuke Yamanoi, Shunta Togo, Yinlai Jiang, Hiroshi Yokoi

**Affiliations:** ^1^Faculty of Informatics and Engineering, The University of Electro-Communications, Tokyo, Japan; ^2^Center for Neuroscience and Biomedical Engineering, The University of Electro-Communications, Tokyo, Japan; ^3^Beijing Advanced Innovation Center for Intelligent Robots and Systems, Beijing Institute of Technology, China

## Abstract

In recent years, myoelectric hands have become multi-degree-of-freedom (DOF) devices, which are controlled via machine learning methods. However, currently, learning data for myoelectric hands are gathered manually and thus tend to be of low quality. Moreover, in the case of infants, gathering accurate learning data is nearly impossible because of the difficulty of communicating with them. Therefore, a method that automatically corrects errors in the learning data is necessary. Myoelectric hands are wearable robots and thus have volumetric and weight constraints that make it infeasible to store large amounts of data or apply complex processing methods. Compared with general machine learning methods such as image processing, those for myoelectric hands have limitations on the data storage, although the amount of data to be processed is quite large. If we can use this huge amount of processing data to correct the learning data without storing the processing data, the machine learning performance is expected to improve. We then propose a method for correcting the learning data through utilisation of the signals acquired during the use of the myoelectric hand. The proposed method is inspired by “survival of the fittest.” The effectiveness of the method was verified through offline analysis. The method reduced the amount of learning data and learning time by approximately a factor of 10 while maintaining classification rates. The classification rates improved for one participant but slightly deteriorated on average among all participants. To solve this problem, verifying the method via interactive learning will be necessary in the future.

## 1. Introduction

For the many amputees living worldwide, various technologies have been developed to support them in their daily lives. One of these technologies is the myoelectric hand, which is used to reconstruct the upper limb functions of amputees. A myoelectric hand functions through the use of biological signals called electromyograms (EMGs), which occur when muscles contract. The myoelectric hand measures these signals to guess the intended hand motion of the wearer and controls the robot hand based on the intention determined by the system.

Approximately half a century ago, myoelectric hands were single-degree-of-freedom (DOF) devices and had several limitations to their hand functions. To counteract these limitations, multi-DOF hands have recently been developed in a number of countries. For example, the BeBionic [1], manufactured by RSL Steeper and acquired by Ottobock in 2017, and i-Limb [2], manufactured by Touch Bionics (which was acquired by Össur in 2016), are well-known multi-DOF myoelectric hands available in the market today. With regard to research endeavours, the SmartHand project [3, 4] funded by the European Commission (EC) aims at developing an intelligent artificial hand that looks and feels like a real hand. One of the products of this prominent project is the SSSA-MyHand [5]. Meanwhile, our group has been developing multi-DOF myoelectric hands for several years now [6–8].

However, despite these advancements, and even with the commercial availability of multi-DOF myoelectric hands, a huge gap between myoelectric hands and human biology remains. It is difficult to duplicate the complex natural function of a human hand via artificial mechanics. The most important and biggest problem that needs to be resolved for multi-DOF robot hands is how to control its multiple actuators using limited EMG signals.

Methods of controlling a multi-DOF myoelectric hand can be divided into three main categories: state transition control, proportional control, and pattern recognition; any method can be based on one, or a combination of more than one, of these categories. The simplest state transition control is known as threshold control, wherein the hand grasp is controlled based on the amplitude of the EMG signal. A more complex form of state transition control, called a finite-state machine, features several states of hand postures and switches to these states via a muscle trigger or physical button. Proportional control is a method that relates the amplitude of an EMG signal to the hand-grasping angle. Some researchers combine state transition control and proportional control to control the posture via state transition and the grasping angle via proportional control. These two methods are intended mainly for the control of a single-DOF myoelectric hand. On the other hand, pattern recognition is intended for use with multiple sensors and therefore for controlling a multi-DOF myoelectric hand. This method mainly uses machine learning, constructs a relationship model between the EMG signals and hand postures, and performs classifications based on the similarity of the input EMG signals to the learned posture model. Compared to the other two methods, pattern recognition can achieve more hand postures through its use of multiple sensors. However, this method requires learning, to construct a model, whenever the myoelectric hand is mounted.

EMG signals are weak and fragile biological signals that vary depending on the individual, and also on daily conditions, even for the same person [7]. Pattern recognition is suitable for detecting minor differences in these EMG signals. Therefore, this method can be used for controlling a multi-DOF myoelectric hand with dexterity because of the capability of machine learning methods to learn and adapt to a given state.

The accuracy of a machine learning method used in pattern recognition depends on the quality of the data used for learning. The general flow of the control of a myoelectric hand via pattern recognition is as follows:

1. The subject wears the myoelectric hand
2. The subject demonstrates a specific muscle activity, and the signals are stored with a label for the corresponding posture
3. Steps 1–2 are repeated for every pattern that needs to be learned
4. After all the necessary data are stored, the classifier learns from these input data
5. After learning, the classifier is now able to classify the current muscle activity using learned parameters and to control the robot hand based on the classification result

In step 2, the labels are assigned by either the subject or an assistant using an external device (e.g., a tablet). However, unlike in general pattern recognition such as image processing, in the case of a myoelectric hand, we do not know if the labels on the learning data are correct or not. Mathematically speaking, the intraclass variance should be small, the interclass variance should be large, and the boundary data should be removed. However, because people manually gather the learning data, they often label very similar muscle activities with different postures, making classification difficult. This activity is especially complicated in the case of infants, with whom it is very difficult to communicate and for whom it is nearly impossible to gather valid learning datasets. Thus, the pattern recognition method is rarely used for infants. We have applied myoelectric hands for infants and children in a clinical setting [9]. The reason why it is difficult to apply pattern recognition methods to infants is a difficulty to communicate with infants and consequently a difficulty to label the signals. However, if we can label the signal appropriately, pattern recognition methods are very useful because they determine classification boundaries as appropriate based on individual muscular activities even if the target is an infant whose muscular activity is unknown. Therefore, we want to semiautomate the labelling signals by the proposed method.

Moreover, EMG signals have time-variant characteristics, which can be problematic. These signals fluctuate in daily life depending on sweat, muscle fatigue, and changes in grasp scheme, among others.

On the other hand, a myoelectric hand has volumetric and weight constraints, and thus, it is infeasible to use a complex discrimination method or to store large amounts of learning data in it. In our previous study, the myoelectric hand stored approximately only 60 to 120 learning data points for three posture classifications, whereas it processed 100 data points per second. If the myoelectric hand is used for an hour, approximately 360,000 data points will be processed, whereas the size of the stored data remains at approximately only 60 to 120. If this huge amount of data can be used for evaluating the stored learning data without having to be stored, the accuracy of the method for controlling the myoelectric hand is expected to improve.

Our objective is to construct a method that automatically corrects the stored learning dataset using EMG signals while the myoelectric hand is in use. To this end, this paper explains how to correct the stored learning data and verifies the usefulness of the method.

The proposed method is expected to obtain the optimal feature space without increasing the calculation burden or storage capacity, even for subjects with whom communication is very difficult, such as infants. Moreover, by adapting to small changes due to factors such as sweat or muscle fatigue, this method is expected to solve the time-variance problem.

## 2. Material and Method

We propose a method of correcting the learning data inspired by the principle of “survival of the fittest.” This phrase is used to describe natural selection based on the Darwinian evolutionary theory [10]. This principle involves three elements: environment, individuals, and groups. The environment is ever-changing, wherein unsuitable individuals die, and suitable individuals thrive, resulting in groups evolving adapted to the environment. The relationship between these elements can be regarded as similar to those in pattern recognition. Pattern recognition has three elements: input data (EMG features, in the case of the myoelectric hand), units, and classes. For a myoelectric hand, a class refers to the postural pattern to be classified, whereas a unit refers to several representative values belonging to a class, which are stored feature vectors. Pattern recognition is the process of determining the unit that is most similar to the input data and outputs the class corresponding to that unit. Classifier performance can be improved when units are provided with high intraclass reproducibility and interclass separability. Based on the principle of “survival of the fittest,” if the EMG features are considered analogous to the environment, the units analogous to individuals, and the classes analogous to groups, we can expect to be able to optimise the units to the EMG features by updating them in parallel with the classification of the input data.

The EMG features (analogous to the environment) depend on the intention of the subject. The subject would not want to use feature space areas where several classes are mixed and would prefer to use independent areas instead, because the expression of muscle activity in mixed areas leads to instability in the control of the myoelectric hand. Therefore, EMG features analogous to the environment encourage evolution toward class separation.

The proposed method is a way of correcting learning data, and thus, it can be combined with all machine learning methods to improve their accuracies.

**Figure 1:**
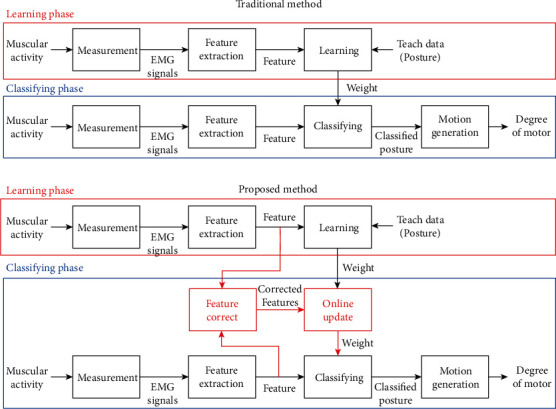
Comparison of processing flow between traditional and proposed methods.

The process flow of the proposed method is illustrated in Figure 1. Typically, machine learning constructs a model using learning data in a learning phase and then classifies further input data using the learned model in a classification phase. By comparison, the proposed method learns the model in the same way and then evaluates the learning data using the data obtained while the wearer controls the hand and updates the model. Because the method uses a large amount of input data, which are obtained while the wearer controls the hand, to evaluate the learning data without storing the input data, the accuracy is expected to improve while the storage capacity and processing burden are maintained.

### 2.1. EMG Features

EMG features comprise a huge amount of nonstored data. Generally, in traditional methods, these data are used only for classification, except in the learning phase. By contrast, in the proposed method, these features are considered as analogous to the environment and are used for the evaluation of the generation and removal of units. In this way, the accuracy of the classifier can be improved without the device having to store large amounts of data.

The wearer of the myoelectric hand is expected to avoid using areas where multiple classes are mixed and to favour using areas where only a single class exists. Therefore, based on the change in the frequency of use, we can correct the learning data without teaching the input data.

EMG signals are fragile neural signals, and thus, the usual method of handling these data is to use extracted features instead of the original signals for input. In this study, the power spectrum (PS) and mean absolute value (MAV) were employed. EMG features can be classified into time-domain and frequency-domain features, and MAV and PS are representative features of these domains, respectively [11]. Recently, methods that use spatial features, called HD-EMG, measured by tens or hundreds of sensors, have also been invented, but they are still in the experimental stage [12, 13]. In our research studies thus far, these features were traditionally employed and tended to have better classification rates than those of other features. Furthermore, the proposed method can be applied to every type of EMG feature.

In this study, the feature vector **V** consists of MAV and PS.

(1) $$\begin{array}{c} V\equiv \left[\begin{array}{c} f_{\text{FE}}\left(s_{t}^{1}\right)\\ f_{\text{FE}}\left(s_{t}^{2}\right)\\ \vdots \\ f_{\text{FE}}\left(s_{t}^{h}\right)\\ \vdots \\ f_{\text{FE}}\left(s_{t}^{N_{\text{ch}}}\right) \end{array}\right],\\ f_{\text{FE}}\left(s\right)\equiv \left[\begin{array}{c} f_{\text{MAV}}\left(s\right)\\ f_{\text{PS}}\left(s\right) \end{array}\right]. \end{array}$$

Here, *s*_*t*_^*h*^ represents the signal sequence on time *t* and the sensor channel *h*, whereas *N*_ch_ denotes the number of sensors. The feature *f*_FE_(*s*) for one sensor has one MAV and several PSs with multiple frequency bands.

MAV is the amplitude of the EMG signals and is defined as follows:

(2) $$\begin{array}{c} f_{\text{MAV}}\left(s_{t}^{h}\right)=\frac{1}{N_{\text{FL}}}\overset{t}{\underset{\tau =t-N_{\text{FL}}+1}{\sum }}\left|s^{h}\left(\tau \right)-{\bar{s}}_{h}\right|,\\ {\bar{s}}_{h}=\frac{1}{N_{\text{FL}}}\overset{t}{\underset{\tau =t-N_{\text{FL}}+1}{\sum }}s^{h}\left(\tau \right). \end{array}$$

Here, *N*_FL_ is the frame length used to extract the feature. In the experiment, *N*_FL_ was 512, and the sampling frequency was 1600 Hz; therefore, the MAV was calculated using the last 0.32 s of data.

On the other hand, PS is the frequency-domain feature and is calculated using the fast Fourier transform (FFT). PS is the magnitude of the energy at a given frequency of the input signal. Several PSs were calculated from a single signal sequence. In this study, PS was calculated as the energy of the frequency band based on the smoothing of neighbouring values.

(3) $$\begin{array}{c} f_{\text{PS}}\left(s_{t}^{h}\right)\equiv \left[\begin{array}{c} f_{\text{PS}}^{1}\left(s_{t}^{h}\right)\\ f_{\text{PS}}^{2}\left(s_{t}^{h}\right)\\ \vdots \\ f_{\text{PS}}^{k}\left(s_{t}^{h}\right)\\ \vdots \\ f_{\text{PS}}^{N_{\text{spct}}}\left(s_{t}^{h}\right) \end{array}\right], \end{array}$$

(4) $$\begin{array}{c} f_{\text{PS}}^{k}\left(s_{t}^{h}\right)=\frac{1}{2I_{P}+1}\overset{k+I_{P}}{\underset{n=k-I_{P}}{\sum }}\left|F\left(n,s_{t}^{h}\right)\right|, \end{array}$$

(5) $$\begin{array}{c} \begin{array}{c} F\left(n,s_{t}^{h}\right)=\frac{1}{N_{\text{FL}}}\overset{N_{\text{FL}}}{\underset{m=1}{\sum }}w\left(m\right)\cdot s^{h}\left(t-N_{\text{FL}}+m\right)\cdot \exp\left\{-j\frac{2\pi }{N_{FL}}n\left(m-1\right)\right\},\\ \text{where }n=0,1,\cdots ,N_{\text{FL}}-1, \end{array} \end{array}$$

(6) $$\begin{array}{c} w\left(m\right)=\exp\left\{-2.0{\left(4.24\times \frac{m}{N_{\text{FL}}}-0.5\right)}^{2}\right\}. \end{array}$$

Here, *I*_*P*_ is the smoothing range and *j* is an imaginary unit. *N*_spct_ is the number of sampling frequency bands per channel. *N*_FL_ is common to the MAV, and the frame is shifted at regular intervals. The assumption is that, before FFT can be applied, the signal must be periodic and continuous. (6) is a window function that makes the signal continuous. Discontinuities in a signal cause errors in the high-frequency components, and thus, we need to calculate up to twice the frequency of PS and use only lower ones. Moreover, Shannon's sampling theorem requires that we sample the data at twice the frequency of the signal [14]. Therefore, the sampling frequency should be four times the maximum frequency of the EMG signals (approximately 10 – 400 Hz).

In this paper, 8 PSs at equal intervals were extracted per channel on the range from 0 to 400 Hz, and the smoothing range was 5. Thus, the extracted frequency bands were 20 – 30, 70 – 80, 120 – 130, 170 – 180, 220 – 230, 270 – 280, 320 – 330, and 370 – 380 Hz.

### 2.2. Units

**Figure 2:**
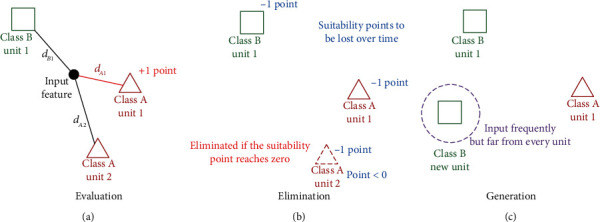
Adaptation of units to input features: (a) evaluation: nearest unit to input feature earns a suitability point; (b) elimination: suitability point of each unit to be lost over time; if it reaches zero, unit is eliminated; (c) generation: if features are inputted frequently far from every unit, new unit belonging to the nearest class is generated.

In the proposed method, units refer to the stored learning data, which are typical data comprising EMG features for each posture. Based on the analogy that the units correspond to individuals in the principle of “survival of the fittest,” the classes are optimised via mooring of those that are suited to the environment and elimination of those that are not. The adaptation of units to EMG features can be expressed in three functions, as shown in Figure 2.

Each unit has a corresponding number of suitability points. When a feature is inputted, the nearest unit earns a suitability point. Units with more suitability points are weighted as more important during the learning of the classifier. The points will be lost over time, and units with points lower than zero are eliminated. Thus, the units placed where data are frequently inputted survive, whereas the units placed where data are rarely inputted are removed, similar to a survival race among organisms.

The calculation for the nearest unit was based on distance. The distance *d*_*u*_*i*__ between the input feature vector **V**_in_ and a unit **u**_*i*_ is calculated using the root sum squares of the difference for each dimension.

(7) $$\begin{array}{c} d_{u_{i}}=\sqrt{\overset{N_{\dim}}{\underset{j=1}{\sum }}{\left\{q_{V_{\text{in}}}^{j}-q_{u_{i}}^{j}\right\}}^{2}}. \end{array}$$

Here, *q* is a feature value, and *N*_dim_ is the dimension size of the feature vectors.

There is no guarantee that the correspondence between the attributes of the unit and the intention of the wearer will be correct. However, it is expected that the wearer will try to change the scheme of muscular contractions if the hand posture is misclassified. In other words, the environment (input EMG features) will change to avoid the misclassification area. As a result, it is expected that an appropriate feature space will be constructed.

Generally, the unit closest to the input data is given a suitability point; however, if the distance between the input data and the closest unit is greater than a certain threshold, no unit can earn the point; furthermore, the coordinates are stored. If data that do not belong to any postures are frequently inputted within a short time and within a close range, the centre of the data is generated as a new unit. This unit is then considered to belong to the nearest class.

### 2.3. Classes

Each class consists of units and corresponds to a posture of the hand. However, even for a single hand posture, muscular activities often differ depending on the force or balance of muscular contractions. It is difficult for anyone, especially an amputee, to maintain constant muscular activity. Thus, it is necessary for a single posture of the myoelectric hand to be expressed in terms of a few different EMG patterns. In this method, because the class has several units, it is possible to assign multiple EMG patterns to the same posture as initial values. Through the optimisation of the feature space, the EMG patterns converge to an easy-to-use placement that is difficult to confuse with another posture. The creation and removal of classes depend on the creation and removal of units that belong to each class.

### 2.4. Manual Correction

If a misclassification occurs early in the learning phase, the optimisation of the feature space will proceed based on a false assessment. Because this method assumes that the feature space does not change drastically, it will be difficult to converge properly once the feature space is significantly broken. Therefore, a function that allows for the manual addition of learning data is necessary. If the usual functionality for gathering learning data can be used in the evaluation phase, the convergence of the feature space can be corrected to a suitable form. Specifically, when the wearer feels that the robot hand is not moving as intended, additional learning data can be gathered using an external device, such as a tablet, in the same way as when the initial learning data were gathered.

### 2.5. Process Flow of Correction, Learning, and Classification in Application

When the proposed method is applied to the myoelectric hand, the first step is for the learning data to be gathered in the same manner as in the traditional method. The EMG features are labelled with corresponding postures using an external device, such as a tablet, and stored. However, because these data are manually gathered, they are likely to contain errors. This is especially true for when the myoelectric hand is being remounted, wherein signals often change because of sensor placement, sweat, and grasping strategies, among others. Therefore, after the hand is remounted, the proposed method corrects the learning data using evaluation data. These evaluation data have no labels for posture, and thus, the learning data can be corrected semiautomatically. Subsequently, the classifier learns the relationship between the EMG features and postures using the extracted data, which comprise learning data already corrected using the evaluation data. After the classifier has finished learning, data to be classified, i.e., without labels for posture, are inputted, and the features are classified into postures.

## 3. Experiment

### 3.1. Overview

The proposed method requires interactions with the wearer. However, comparisons of performance with and without the new method can be difficult to perform. Therefore, we conducted a basic offline analysis. As described previously, the feature space will converge incorrectly if misclassification occurs early in the learning phase. For this reason, we verified the evaluation and removal functions, excluding the generation functions.

A dataset that was used in previous research [15] was employed. This dataset was collected to validate the robustness of methods to several kinds of changes in EMG signals: grasping force, postures, subjects, remounting of sensors, and repetition.

#### 3.1.1. Subjects

The subjects were three healthy males in their twenties. Two of them were right-handed, and one was left-handed. For all subjects, the signals were measured from the right forearms.

#### 3.1.2. Task

The raw EMG signals, PS, and average MAV were displayed for the subjects. They were instructed to change their grasping forces and postures to follow the target waveforms. The nine target postures were as follows: rest (rst), open palm (opn), power grasp (pwg), precision grasp (prg), lateral grasp (ltg), wrist flexion (flx), wrist extension (ext), wrist pronation (prn), and wrist supination (spn). These three grasping postures (pwg, prg, and ltg) are called basic types of hand postures because 80% of the activities of daily living (ADL) can be achieved with these postures [5]. Furthermore, the target grasping force was set to three levels for each posture, except for the rest posture. Controlling the grasping force prevents a difference in force levels from corresponding to a difference in posture patterns.

#### 3.1.3. EMG Measurement and Preprocessing

The EMG signals were measured using sensors that we have developed. First, a sensor measured the signal while the in-phase component of the noise was removed using a differential amplifier. Power line interference (PLI) at approximately 50 Hz was then removed using a notch filter, and only the frequency band of the EMG signals was extracted using a bandpass filter (10–400 Hz). Finally, a secondary amplifier was used to amplify the signal. The signal was amplified by approximately 80,000 times in total using the differential amplifier and secondary amplifier. The sampling frequency was 1600 Hz.

#### 3.1.4. Placement of Sensors

**Figure 3:**
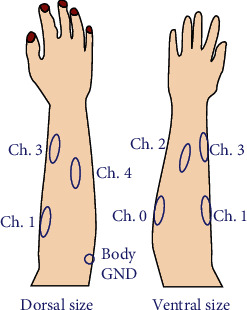
Placement of sensors.

Five sensors were employed for this experiment. These sensors were attached to the flexor digitorum superficialis (ch. 0), extensor digitorum (ch. 1), abductor pollicis longus (ch. 2), extensor indicis (ch. 3), and extensor digiti minimi (ch. 4), as shown in Figure 3. These muscles meet the three requirements for myoelectric hands: located in the forearm, related to the movement of the fingers, and can be determined via external palpation [16].

#### 3.1.5. Remounting of Sensors

The EMG signals were measured in nine trials against a day equivalent. Nine postures were included in the trial, and the target force was decided quasirandomly. “Quasirandomly” signifies that the order is random, but the number of appearances for each event is equal. As a result, there were three trials for each target force and posture in a single dataset against a day equivalent. Subsequent measurements were obtained after sufficient time had passed since the removal of the sensor. Multiple measurements were performed over several days, and task achievement levels were calculated. A task achievement level refers to how much the measured data deviated from the target data; a smaller value is considered to be more desirable. The three-day equivalent data with the lowest task achievement levels for each subject were then selected for further analysis.

### 3.2. Classifier

**Figure 4:**
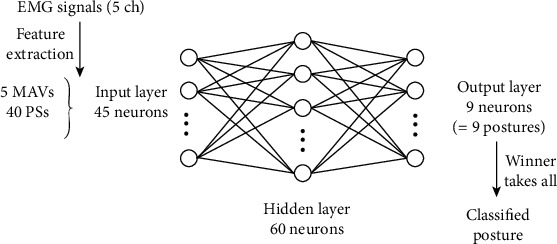
Structure of ANN (three-layered feed-forward type).

Both the proposed and traditional methods employ a three-layered feed-forward type artificial neural network (ANN) as the classifier. The structure of the ANN is shown in Figure 4. In this study, the hyperparameters were the same as those shown in Table 1. The dimension of the input was 45, which consisted of five MAV and 40 PS from five channels. The output was a value between 0 and 1 for each posture; the posture of the neuron with the highest value was regarded as the classified posture, or the posture resulting from the classification, based on the principle of “winner takes all.” The proposed method performed weighted learning based on suitability points. Furthermore, the threshold to determine the nearest unit was set to infinity, and thus, there was never a situation where no unit earned a suitable point. Therefore, no additional units were generated.

### 3.3. Validation

The dataset of a day equivalent consisted of nine trials. It was divided into three parts such that the posture and force conditions were equal, and cross-validation was performed. One part of day X was used for learning, one part of day Y was used for classification, and the other two parts of day Y were used for evaluation. Classification rates were calculated for all combinations of days.

Although using datasets from different days for learning and classification, i.e., with the myoelectric hand remounted, reduces the classification rates, the proposed method is expected to improve the classification rates using input data, which are obtained from the myoelectric hand after remounting, for evaluation.

The effectiveness of the proposed method was validated based on comparisons in terms of the number of learning data points, learning time, and classification rates.

## 4. Result and Discussion

### 4.1. Number of Learning Data Points

The numbers of learning data points for both the traditional and proposed methods are shown in Table 2. The traditional method constructed a relationship model between the EMG signals and hand postures using all of the learning data. By contrast, in the proposed method, the learning data were scored using the evaluation data, and most of them were removed. The proposed method then constructed a model using the extracted data that survived. As shown in Table 2, the ratios of extracted data for each subject were 6.4% to 7.6%.

Verification was performed using a laptop PC, which is capable of storing much more data than a myoelectric hand is capable of storing in real-life application. The size of the extracted data is reasonable for a microcomputer to store and process it.

As mentioned earlier, this research involved nine target postures, and the target grasping force was set to three levels for each posture, excluding the rest posture. Hence, 25 types of muscle activities were assigned to the classifier as input. The number of extracted data points per type was approximately 15, which is a very small number compared with those used in previous studies. As shown later, the classification rates were maintained, and thus, the feature space was adequately represented using a small amount of data. Because myoelectric hands have weight and volume constraints, it is important to reduce the amount of data to be stored.

### 4.2. Learning Time

The learning times of both the proposed and traditional methods are listed in Table 3. The learning time is the time used to construct the model, which includes the time to load the data and to output the result. The load and output times were sufficiently short compared to the learning time. In the traditional method, learning was performed based on all the learning data, whereas in the proposed method, learning was performed based on only the extracted data. If the learning cycle exceeded the maximum learning time (20,000 cycles), or if the error per neuron was below the determined truncation error (0.0002), the learning was considered completed. As shown in the table, the learning time of the proposed method is significantly reduced. The main reason for the decrease in learning time was the reduction in the number of learning data points. As mentioned previously, reducing the processing burden is important for myoelectric hands.

### 4.3. Classification Rates

**Figure 5:**
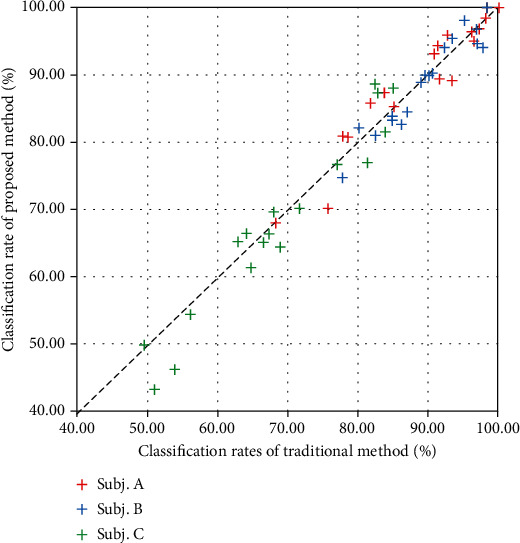
Classification rates of the proposed method against the traditional method (subjects are represented by colours). If the plot is above the centre line, it indicates improved classification rates; if the plot is below the centre line, it indicates deteriorated classification rates.

The classification rates for each subject are shown in Table 4, and a comparison between the classification rates of the traditional and proposed methods is shown in Figure 5.

As a result of the proposed method, based on the averages, the classification rates decreased slightly. It should be noted that the numbers of data points used for learning were distinctly different, and thus, this comparison was unfavourable to the proposed method. According to the details of each trial, there were cases wherein the proposed method worked well and wherein it did not.

**Figure 6:**
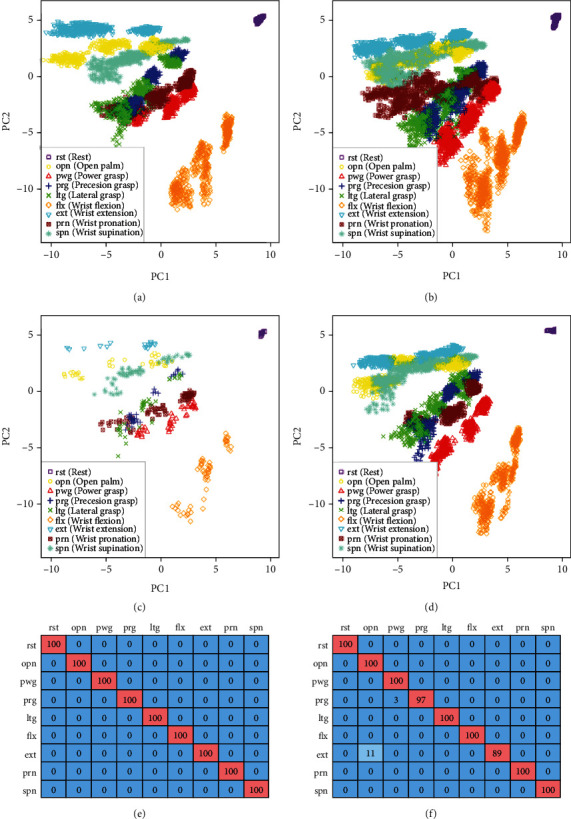
Case where the proposed method works well. Axes are common and calculated via principal component analysis (PCA) using learning data. PC1 and PC2 denote first and second principal components, respectively. Specific values are meaningless in (a) to (d) because whitening is performed during PCA. (a) Learning data (Subj. A Day 2 part. 3). (b) Evaluation data (Subj. A Day 1 part. 1, 2). (c) Extracted data (Subj. A Day 2 part. 3). (d) Classifying data (Subj. A Day 1 part. 3). (e) Classification rate of the proposed method (Ave. 100.00%). (f) Classification rate of the traditional method (Ave. 98.44%).

An example of a case in which the proposed method worked well is shown in Figure 6. In Figures 6(a)–6(d), the feature spaces were dimensionally compressed into two dimensions via principal component analysis (PCA). The axis scales of the images were unified. Each posture was placed radially around the rest posture, and the stronger the force, the further out it was placed. They were also placed in the flexor and extensor muscle groups. Subfigures (e) and (f) show confusion matrices, wherein the rows indicate actual postures, and the columns indicate classified postures. Some adjacent postures were misclassified by the traditional method, e.g., open palm and wrist extension. By contrast, in the proposed method, the extracted data that were selected were only the characteristic data similar to the evaluation data, resulting in improved classification rates.

However, as stated earlier, there were also cases in which the proposed method did not work well. There are two main causes for these cases. First, if the classification rate of the traditional method is excessively low, the proposed method will not work well because it is assumed that the feature space does not change drastically. The low classification rates of the traditional method indicated that the muscle activities were not reproducible.  Table 5 summarises the trend of improvement or deterioration of the classification rate of the proposed method compared to that of the traditional method. Trials in which the classification rate increased by more than 1% were defined as “improved,” those that decreased by more than 1% were defined as “deteriorated,” and the others were defined as resulting in a “small difference.” Figure 5 and Table 5 show that when the classification rates of the traditional method were high, “improved” tended to be more frequent, whereas when the rates were low, “deteriorated” tended to be more frequent. The difficulty of the task performed in this experiment was high because of the large number of target postures and force levels. In actual applications, an appropriate number of postures should be set according to the subject, and the number of postures should be increased step by step through training.

**Figure 7:**
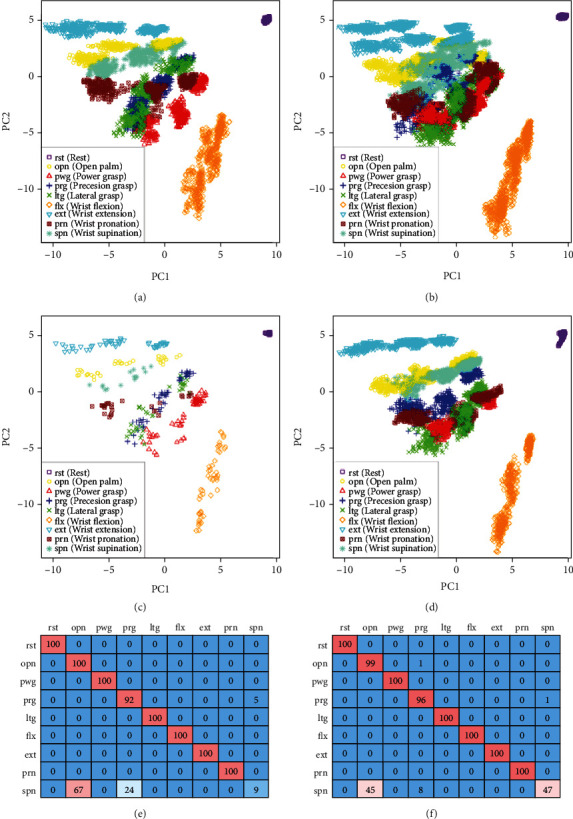
Case where posture was misclassified. Representations of figures are the same as in Figure 6. (a) Learning data (Subj. A Day2 part. 1). (b) Evaluation data (Subj. A Day 3 part. 2, 3). (c) Extracted data (Subj. A Day 2 part. 1). (d) Classifying data (Subj. A Day 3 part. 1). (e) Classification rate of the proposed method (Ave. 89.01%). (f) Classification rate of the traditional method (Ave. 93.51%).

The second main cause of the proposed method not working very well is that there were misclassifications of certain postures. These were observed when the classification rates of the traditional method were high but were deteriorated by the proposed method. An example of a case wherein certain postures were misclassified is shown in Figure 7. The representation in Figure 7 is the same as that used in Figure 6. Based on the feature space of the evaluation data, the repeatability of wrist supination was low and became confused with the open palm and precision grasp. As a result, in the feature space of the extracted data, only a small number of units that take responsibility for wrist supination have survived. This signifies that, in the evaluation phase, suitability points were given for units of incorrect postures. As a result, the classification rate of wrist supination was significantly reduced.

In this study, we performed an offline analysis; however, to truly solve this problem, we need to verify the effectiveness of our proposed method when used together with interactive learning and unit generation functionalities.

## 5. Conclusion

In machine learning, the quality of learning data is very important. However, because of the volumetric and weight constraints of myoelectric hands, the limited storage capacity and processing performance should also be considered. Therefore, we aimed to construct a method for automatically correcting the stored learning dataset using EMG signals while the myoelectric hand is in use. Our proposed method is inspired by the principle “survival of the fittest.” Partial verification of the proposed method via offline analysis showed that it reduced the number of learning data points and learning time while maintaining the classification rates. Although the classification rates improved for one participant, they slightly deteriorated on average among all participants. To solve this problem, it is necessary to verify the method when used together with interactive learning. We intend to perform that study in the near future.

## Figures and Tables

**Table 1:** Hyperparameters of a classifier.

Hidden neuron	60
Learning rate	0.0002
Learning times	20,000
Initial value	−0.1 to 0.1

**Table 2:** Number of extracted data points.

	Extracted (proposed)	Learning (traditional)	Ratio
Subj. A	414.1 ± 57.2	5413.0 ± 1.7	7.6%
Subj. B	369.7 ± 38.7	5411.7 ± 2.7	6.8%
Subj. C	346.4 ± 57.8	5412.0 ± 3.0	6.4%
Ave.	376.7 ± 58.3	5412.2 ± 2.5	7.0%

**Table 3:** Learning time (min:s).

	Proposed	Traditional	Ratio
Subj. A	1 : 18 ± 0 : 09	10 : 57 ± 0 : 09	11.9%
Subj. B	1 : 10 ± 0 : 08	10 : 16 ± 2 : 22	11.4%
Subj. C	1 : 09 ± 0 : 09	11 : 09 ± 0 : 06	10.3%
Ave.	1 : 12 ± 0 : 10	10 : 47 ± 1 : 24	11.3%

**Table 4:** Classification rates (%).

	Proposed	Traditional	Difference
Subj. A	89.24	88.80	+0.44
Subj. B	67.87	68.15	−0.29
Subj. C	89.08	89.69	−0.61
Ave.	82.06	82.21	−0.15

**Table 5:** Number of datasets improved or deteriorated by the proposed method.

	Classification rate of the traditional method			
	100 to 90	90 to 80	80 to 70	70 to 0
Improved (more than 1%)	8	6	2	3
Small difference (−1% to 1%)	7	3	1	3
Deteriorated (less than −1%)	5	7	3	6
